# Are face recognition abilities in humans and sheep really ‘comparable’?

**DOI:** 10.1098/rsos.180772

**Published:** 2019-01-23

**Authors:** A. Towler, R. I. Kemp, V. Bruce, A. M. Burton, J. D. Dunn, D. White

**Affiliations:** 1 School of Psychology, The University of New South Wales, Sydney, Australia; 2 School of Psychology, Newcastle University, Newcastle upon Tyne, UK; 3 Department of Psychology, University of York, York, UK

Knolle *et al*. [1] tested the ability of sheep to recognize human faces. Their results provide a compelling demonstration of advanced human-face recognition abilities in sheep, and make an important contribution to work seeking to understand the visual, cognitive and neural processes driving face and object recognition in non-human animals. However, as scientists who study face recognition in humans, we believe Knolle *et al*.'s claim that ‘sheep have advanced face-recognition abilities, *comparable with those of humans*' (abstract, page 1) is unwarranted given their data.

In support of comparable abilities, Knolle *et al*. cite a 15% drop in human-face recognition performance from frontal to changed views of faces in both sheep and humans, and the fact that sheep recognize a familiar human handler above chance levels. By ‘comparable’, Knolle *et al*. may mean that sheep and humans have comparable levels of *performance* on human-face recognition tasks, or that sheep and humans show comparable *patterns* of performance on human-face recognition tasks and thus share similar mechanisms for recognizing human faces.

Below we outline why neither of these interpretations are justified: (i) the absence of human performance data on an equivalent task prevents meaningful comparison between sheep and humans; (ii) unfamiliar and familiar human-face recognition accuracy reported in their study is substantially lower than would be expected for humans; and (iii) if anything, their data suggest that sheep and humans recognize human faces using qualitatively different mechanisms.

In Knolle *et al*.'s study, sheep learned to recognize four unfamiliar human faces by association with a reward. Sheep learned the face–reward associations from a frontal face photograph across three training blocks of increasing difficulty. In one test condition, sheep were shown pairs of images, one of which was identical to one of the four learned images and the other was a novel unfamiliar face randomly selected from a pool of gender and ethnicity matched faces. In this identical-image condition, sheep chose the learned face over the novel face on 79% of trials. In a novel-image condition, the experimenters showed sheep a novel image of the learned identity, taken on a different day and from a different angle. Remarkably, sheep chose the learned identity over the novel identity on 66% of trials, significantly better than the 50% expected by random responding.

Recognizing learned identities from novel images, where factors such as hairstyle, makeup, health, lighting and camera differ across photographs, is challenging even for humans, and the ability to recognize an identity despite this ‘within-face’ variability is a hallmark of familiarity [2]. The fact that sheep recognized learned identities from novel images taken on different occasions above chance is, therefore, the most compelling direct demonstration of genuine human-face recognition abilities in non-primate animals that we have seen (see also [3]). Almost all previous human-face recognition research in animals, such as sheep, honeybees, dogs and archerfish, has used *identical* images at training and test (e.g. [4–7], but see [8]). These studies therefore measure *image* recognition, which can be solved using low-level visual pattern-matching strategies, rather than *face* recognition *per se* [9,10] (see also [11]).

We acknowledge that Knolle *et al*. have shown that, like humans, sheep are capable of recognizing a *person* in an image, not just an image. However, to conclude that sheep have *comparable* face recognition abilities to humans, it is necessary to compare sheep and human performance on an equivalent task. Knolle *et al*. compared sheep performance to human recognition accuracy scores from Bruce [9]. However, Knolle *et al*.'s experimental paradigm differs from Bruce's recognition task in some key details. First, sheep were only required to learn four identities, whereas Bruce required human participants to study 24. Second, Knolle *et al*. trained sheep to threshold over three identity-learning blocks over several days, whereas participants in Bruce's study saw each identity just once for 8 s. Third, sheep selected which of two images showed the learned identity, whereas Bruce employed a more challenging ‘old–new’ memory test, where faces were presented sequentially and participants decided whether or not each face had been presented in the learning phase.

We do not know of any human studies directly comparable to Knolle *et al*.'s methodology. However, data on human-face recognition in humans are extensive, and show that humans typically attain accuracy well above that achieved by sheep, on recognition memory tasks that are more complex and more demanding than that described by Knolle *et al*. For example, Longmore *et al*. [12] found that after learning 12 unfamiliar identities from single photographs with feedback, participants recognized the learned identities in a novel pose in an old–new paradigm with 85% accuracy. In another condition, after a single 5 s exposure to each of the 12 faces, participants recognized those identities in a novel pose with 70% accuracy. Given that human participants achieved higher levels of performance when remembering larger sets of faces, and with far less exposure, it is reasonable to infer that human performance given the task parameters used by Knolle *et al*. would far exceed 66%.

Rather than basing their conclusions on the absolute accuracy of sheep in their task, Knolle *et al.*'s conclusions may be based on qualitative similarities between sheep and humans—i.e. *patterns* of performance. For example, in support of comparable abilities, Knolle *et al*. cite a 15% drop in recognition performance from frontal to changed views of faces in both sheep and humans. However, without human performance data on a comparable task it is impossible to know that these reductions in accuracy are equivalent. Further, even if data were to show equivalent reductions in accuracy as a function of pose change, it is still possible that different underlying mechanisms produce these behavioural similarities. For example, face recognition algorithms that use very different approaches can nevertheless show similar sensitivity to pose changes [13].

Indeed, aspects of Knolle *et al*.'s data suggest that sheep and humans use qualitatively *different* mechanisms to recognize human faces. For example, sheep and humans appear to possess different *familiar* human-face recognition abilities. In the test phase, sheep performed three trials where a high-quality frontal photo of a highly familiar handler was paired with a gender and ethnicity matched unfamiliar face. Sheep chose the handler's face over the unfamiliar face on 72% of trials. Importantly, sheep recognized this highly familiar face with the same level of accuracy as newly learned identities (68%). This stands in contrast to a large literature showing that humans recognize familiar faces with far higher accuracy compared with recently learned unfamiliar faces [14], and that we can recognize familiar people with near perfect accuracy even in very challenging viewing conditions [15]. The fact that sheep did not show better recognition of the familiar handler suggests that human-face recognition in sheep is not reliant on human-like face processing mechanisms.

Interestingly, some studies suggest qualitative similarities between how sheep recognize *sheep* and how humans recognize humans. For example, sheep and humans both show face inversion effects [16–19], selective neural activation for familiar conspecifics [17,20] and tend to recognize familiar conspecifics using internal rather than external features [21,22]. These findings may suggest that social pressure to recognize the faces of conspecifics led to the development of specialized face recognition mechanisms in both species. The extent to which these mechanisms overlap and generalize to other species is a fascinating topic for future study.

Critically however, no studies that we are aware of include a human participant comparison group, despite several papers making claims about the comparability of sheep and human face recognition [4,23]. Without human performance data on an equivalent task, there is very little basis upon which to make claims about the similarity of the mechanisms underlying human-face or conspecific-face recognition in sheep and humans.

## Data accessibility

This article has no additional data.
